# Cynaropicrin: A Comprehensive Research Review and Therapeutic Potential As an Anti-Hepatitis C Virus Agent

**DOI:** 10.3389/fphar.2016.00472

**Published:** 2016-12-08

**Authors:** Mahmoud F. Elsebai, Andrei Mocan, Atanas G. Atanasov

**Affiliations:** ^1^Department of Pharmacognosy, Faculty of Pharmacy, Mansoura UniversityMansoura, Egypt; ^2^Department of Pharmaceutical Botany, Iuliu Haţieganu University of Medicine and PharmacyCluj-Napoca, Romania; ^3^Department of Pharmacognosy, University of ViennaVienna, Austria; ^4^Institute of Genetics and Animal Breeding of the Polish Academy of SciencesJastrzebiec, Poland

**Keywords:** cynaropicrin, anti-hepatitis C virus, anti-hyperlipidemic, antitumor, anti-inflammatory, anti-parasite, antibacterial, anti-gastritis action

## Abstract

The different pharmacologic properties of plants-containing cynaropicrin, especially artichokes, have been known for many centuries. More recently, cynaropicrin exhibited a potential activity against all genotypes of hepatitis C virus (HCV). Cynaropicrin has also shown a wide range of other pharmacologic properties such as anti-hyperlipidemic, anti-trypanosomal, anti-malarial, antifeedant, antispasmodic, anti-photoaging, and anti-tumor action, as well as activation of bitter sensory receptors, and anti-inflammatory properties (e.g., associated with the suppression of the key pro-inflammatory NF-κB pathway). These pharmacological effects are very supportive factors to its outstanding activity against HCV. Structurally, cynaropicrin might be considered as a potential drug candidate, since it has no violations for the rule of five and its water-solubility could allow formulation as therapeutic injections. Moreover, cynaropicrin is a small molecule that can be easily synthesized and as the major constituent of the edible plant artichoke, which has a history of safe dietary use. In summary, cynaropicrin is a promising bioactive natural product that, with minor hit-to-lead optimization, might be developed as a drug for HCV.

## Introduction

**Figure 1:**
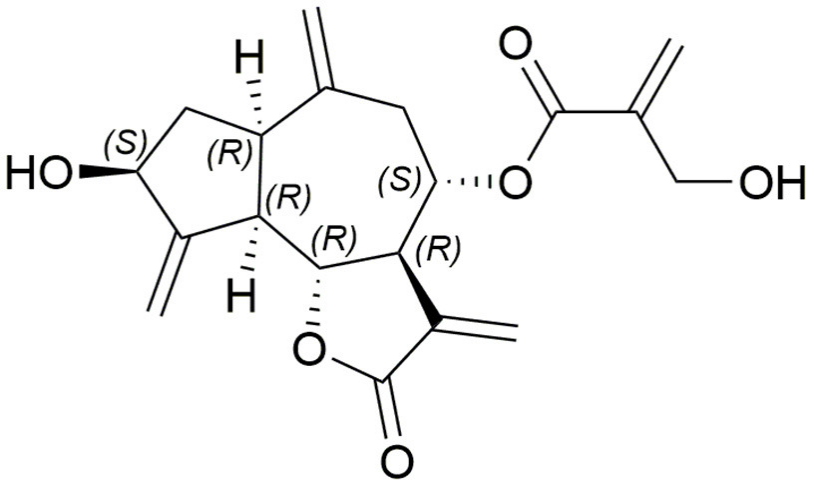
**Structure of cynaropicrin**.

Cynaropicrin (Figure 1) is a sesquiterpene lactone of a guaianolide type. It has a 5-7-5 fused tricyclic skeleton with six stereocenters, four exo-olefins, and two hydroxyl groups. The γ-butyrolactone ring is a very important pharmacophore which is implicated in many biological activities of cynaropicrin. In 1960, cynaropicrin was first isolated from artichoke (*Cynara scolymus* L.) (Suchy et al., 1960) and it is now considered as a chemotaxonomic marker of artichoke plants (Chaturvedi, 2011). The artichoke plants are known to exhibit significant pharmacological effects and health benefits that have recently been reviewed (Ben Salem et al., 2015).

The bitter taste of artichoke plants is attributed to its high content of sesquiterpene lactones, especially of cynaropicrin. Cynaropicrin contributes to approximately 80% of the characteristic bitter taste of artichoke, which is associated with the activation of bitter sensory receptors (Cravotto et al., 2005; Eljounaidi et al., 2015). Cynaropicrin can be isolated in gram-scale by employing countercurrent chromatography (Adekenova et al., 2015). ^1^H NMR spectroscopy and titration of the lactone ring with sodium hydroxide were used for the quantitative determination of cynaropicrin (Schneider and Thiele, 1974a,b; Pieri and Stuppner, 2011).

The chemical resonance peaks in the ^13^C NMR of many sesquiterpene lactones including cynaropicrin and some of its derivatives were reported (Budesinsky and Saman, 1995). The hydroxy pentanoid ring is *cis* fused to the heptanoid ring and the lactone ring has a *transoid* orientation with the heptanoid one. The absolute stereochemistry of cynaropicrin was determined by chemical relation to α-santonin (Corbella et al., 1972).

Cynaropicrin is a sesquiterpene lactone; sesquiterpene lactones are the most biologically significant class of secondary metabolites. Cynaropicrin has been shown to possess various biological activities and has demonstrated extraordinary pharmacologic properties such as anti-hepatitis C virus, anti-parasitic, anti-tumor, anti-hyperlipidemic, antifeedant, antispasmodic, anti-photoaging agent, activation of bitter sensory receptors, suppression of NF-κB, and anti-inflammatory properties.

## Anti-hepatitis C virus (anti-HCV) activity

Human infection with HCV is currently recognized as the leading cause of chronic liver diseases such as hepatic steatosis, liver cirrhosis, and hepatocellular carcinoma (HCC), which demands liver transplantation (Ishida et al., 2014). Hepatitis C virus infection is a significant public health problem with approximately 200 million people around the world being infected with HCV (Tsantrizos, 2008; Ibrahim et al., 2013). About 3–4 million people are infected per year, and the World Health Organization (WHO) reported that approximately 700,000 people die each year from hepatitis C-related liver diseases (http://www.who.int/mediacentre/factsheets/fs164/en/), with the US mortality rates from HCV now exceeding those from HIV. The overall medical and social costs of chronic HCV infections are estimated to exceed $85 billion (Ibrahim et al., 2013).

**Figure 2:**
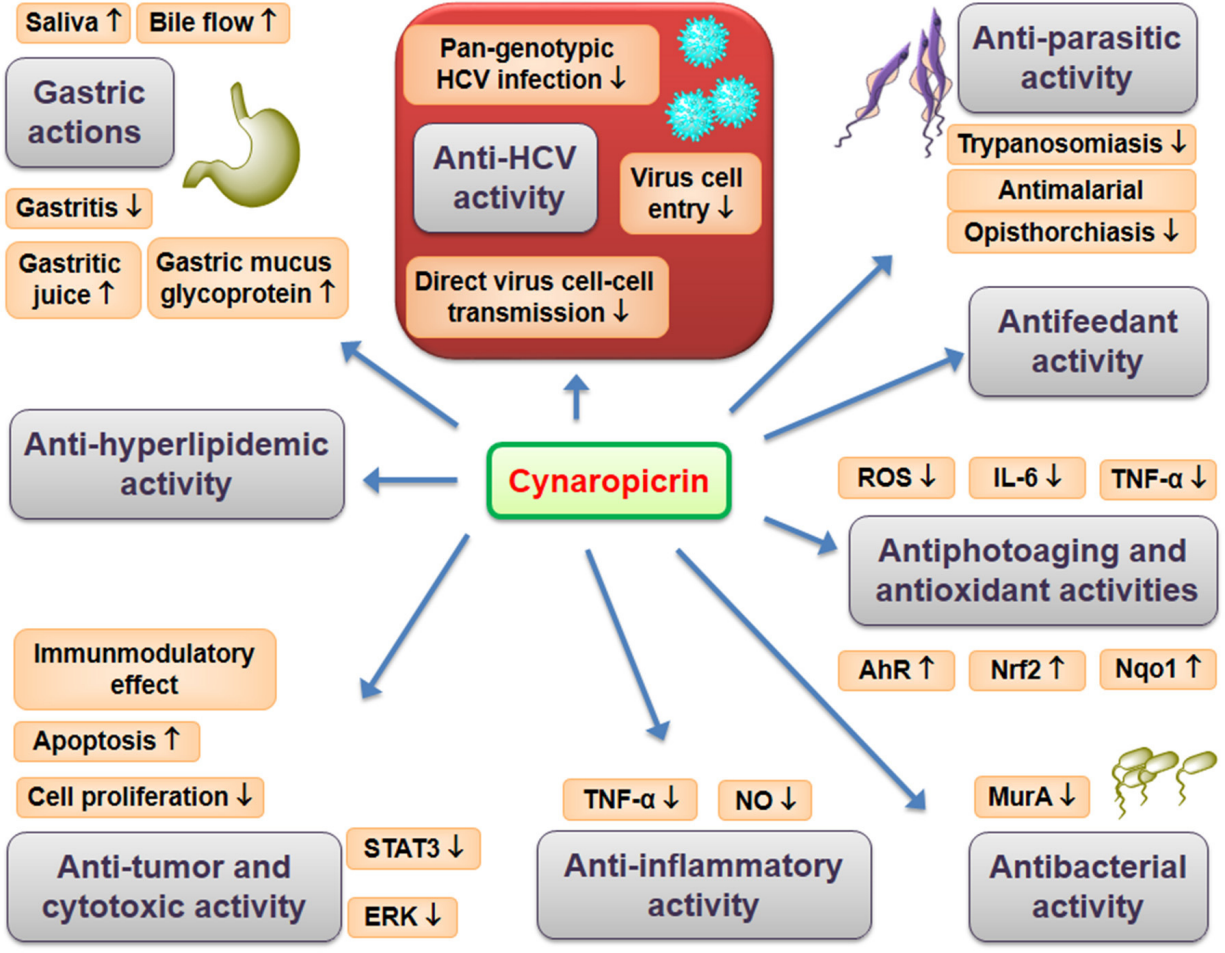
**Pharmacological effects of cynaropicrin**.

The wild Egyptian artichoke exhibited promising activity against HCV (Elsebai et al., 2016a) which were related to its sesquiterpene lactones especially cynaropicrin (Elsebai et al., 2016b). Cynaropicrin demonstrated outstanding activity against HCV since, for the first time, the performed *in vitro* studies showed that cynaropicrin has potent and broad spectrum activity as a cell-entry inhibitor against all genotypes of HCV with EC_50_ in the low micromolar range (Elsebai et al., 2016b). Cynaropicrin acts during the early steps of the HCV lifecycle, including cell-free and cell-cell infection inhibition. HCV is transmitted between hepatocytes via classical cell entry using cell-free diffusion but also uses direct cell-cell transfer to infect neighboring cells. Interestingly, cynaropicrin efficiently inhibited cell-cell transmission, which was confirmed by using a co-culture of two different cell types: Huh7/Scr cells infected with the Jc1 virus act as HCV donor cells while Huh7.5/EGFP-NLS-IPS cells act as acceptor cells. Furthermore, the antiviral activity of cynaropicrin was pan-genotypic as HCV genotypes 1a, 1b, 2b, 3a, 4a, 5a, 6a, and 7a were inhibited. Thus, cynaropicrin is a promising candidate for the development of new and cost-effective pan-genotypic entry inhibitors of HCV infection (Elsebai et al., 2016b). The following pharmacological effects can be linked directly or indirectly with the promising anti-HCV activity of cynaropicrin. Overview of reported pharmacological effects of cynaropicrin is presented on Figure 2.

## Anti-hyperlipidemic activity

The leaf-extract of artichoke (*Cynara scolymus* L.) suppressed serum triglyceride elevation in olive oil-loaded mice. This anti-hyperlipidemic activity might be through the suppression of gastric emptying. Cyanopicrin, aguerin B, and grosheimin were isolated as the anti-hyperlipidemic compounds and the activity of cynaropicrin was the most potent among them. The oxygen functional groups and exo-methylene moiety in α-methylene-γ-butyrolactone ring were essential for the activity of these guaiane-type sesquiterpenes (Shimoda et al., 2003). A set of tricyclic sesquiterpene lactones including cynaropicrin was applied for patenting in the treatment of obesity and related diseases and non-therapeutic treatable conditions (Grothe et al., 2013).

## Anti-tumor and cytotoxic activity

Cho et al. reported that the cynaropicrin is the first naturally occurring compound which modulates the functional activation of major adhesion molecules (CD29 and CD98) in macrophages, using a quantitative aggregation assay established with U937 cells and activating antibodies to their major adhesion molecules (Cho et al., 2004a). The activation of adhesion molecules is an essential factor in regulating inflammatory process. Once activated, immune cells migrate to inflamed tissues and cell-cell adhesion is facilitated (Muller, 2003). Cynaropicrin inhibited CD98- and CD29 (β1 integrins)-induced homotypic aggregation with IC_50_ values of 2.98 and 3.46 μM, respectively, without displaying cytotoxicity. On the contrary, CD43-induced hemolytic aggregation was not inhibited, suggesting that cynaropicrin-induced inhibition is due to a specific immunopharmacological effect. In addition, cynaropicrin regulatory effect on CD29 and CD98 is mediated through the inhibition of extracellular signal-related kinase (ERK) pathway. Cynaropicrin is considered as a potential drug for treatment of CD29- and CD98-mediated diseases such as virus-induced chronic inflammation, and invasion, migration, and metastasis of leukocyte cancer cells (Cho et al., 2004a).

Cynaropicrin may be a potential anticancer agent against some leukocyte cancer cells such as lymphoma or leukemia, through pro-apoptotic activity. The cytotoxic effect of cynaropicrin against macrophage, eosinophil, fibroblast, and lymphocyte cell lines was evaluated. Cynaropicrin showed selective potential antiproliferative activity against differentiated human macrophage (U937 cells), Eol-1 and Jurkat T leukocyte cell lines. Meanwhile, it was not as active against Chang liver cells and human fibroblast cell lines. The mechanism of the cytotoxic effect of cynaropicrin on U937 cells was found to be mediated through induction of apoptosis and cell cycle arrest at G1/S phase. Cynaropicrin cytotoxic activity was inhibited in presence of N-acetyl-L-cysteine and L-cysteine, reactive oxygen species (ROS) scavengers, or rottlerin [protein kinase (PK) Cδ inhibitor]. Therefore, it was concluded that PKCδ and ROS are important for the pro-apoptotic activity of cynaropicrin due to the cynaropicrin-induced proteolytic cleavage of PKCδ (Cho et al., 2004b).

The ethanol extract of the aerial part of the Mongolian medicinal plant *Saussurea salicifolia* induced a dose-dependent cell growth inhibition in both human gastric adenocarcinoma (AGS) cells and mouse hepatoma Hepa 1c1c7 cells (IC_50_ = 30.22 and 116.96 μg/ml, respectively). Seven bioactive compounds causing the apoptosis were isolated including cynaropicrin, three lignans (arctigenin, matairesinol, and trachelogenin) and three lignan glycosides (arctiin, matairesinoside, and tracheloside). Cynaropicrin and arctigenin were the most active and they inhibited the proliferation and induced apoptosis in AGS cells (IC_50_ = 0.68 and 31.90 μg/ml, respectively) in a dose-dependent manner. Thus, cynaropicrin and arctigenin are thought to be responsible for the antiproliferative and pro-apoptotic activity of *S. salicifolia* total extract in AGS cells. Therefore, cynaropicrin may serve as a potential drug lead for treatment or prevention of human cancers (Kang et al., 2007).

Cynaropicrin suppressed IL-6-inducible and constitutive Signal Transducer and Activator of Transcription 3 (STAT3) activation (Butturini et al., 2013). STAT3 is a cytoplasmic transcription protein factor that is activated in various cancers. STAT3 is controlled not only by phosphorylation but also by S-glutathionylation. It inhibits apoptosis, induces chemoresistance, stimulates cell proliferation, and promotes angiogenesis, invasion, and migration. Consequently, STAT3 is considered as a potential cancer therapeutic target through counteracting its hyper-expression or hyper-activation. In the human prostate cancer cell line DU145, that constitutively express active STAT3, STAT3 inhibition led to the suppression of two anti-apoptotic genes, Bcl-2 and denocarc. With an IC_50_ of 12 μM, cynaropicrin inhibited both IL-6-inducible and constitutive STAT3 activation in THP- 1 cells and the cell line DU145. It showed synergistic effects with the chemotherapeutic agents cisplatin or docetaxel. Cynaropicrin induces a rapid drop in intracellular GSH concentration in a dose-dependent manner through Michael addition reaction, thereby triggering S-glutathionylation of STAT3, interfering with its phosphorylation. Cynaropicrin was found to regulate STAT3 function through induction of redox-dependent post-translational modification of STAT3 cysteine residues (Butturini et al., 2013).

Multiple cancer drugs established in clinics act via exhibiting genotoxic effects targeting proliferating cancer cells (Swift and Golsteyn, 2014). The genotoxic potential of cynaropicrin was evaluated using the Homozygotization Index (HI) test on two diploid strains of *Aspergillus nidulans*: UT184//UT448, with normal DNA repair mechanisms; and *Dp* II-I//UT184, presenting recombinational repair mechanisms only. Potential genotoxic/ carcinogenic compounds can be detected by this test at low doses. Treatments of UT448/UT184 and *Dp* II-I/UT184 diploid strains with cynaropicrin at a concentration of 25 μg/ml increased the HI of both strains, especially for the diploid *Dp* II-I/UT184. This is the first time the low dose effect of a bioactive natural product such as cynaropicrin was detected using *A. nidulans* (Salvador et al., 2008).

Cynaropicrin and desacylcynaropricrin (isolated from the Tibetan plant *Saussurea eopygmaea*, Compositae) have *in vitro* activity against both solid and ascites tumors (S-180 sarcoma and Ehrlich carcinoma) with IC_50_ values of 2.5 and 2.4 μg/ml for cynaropicrin and 1.5 and 1 μg/ml for desacylcynaropricrin, respectively (Zong et al., 1994). Cynaropicrin (from the flowers of *Hemisteptia lyrata* Bunge) showed cytotoxic activity against SK-OV-3 (human ovary adenocarcinoma cell), LOX-IMVI (human melanoma cell), A549 (human non-small lung adenocarcinoma cell), MCF-7 (human breast adenocarcinoma cell), PC-3 (human prostate adenocarcinoma cell), and HCT-15 (human colorectal adenocarcinoma cell) cell lines with IC_50_ values between 1.1 and 8.7 μg/ml (Ha et al., 2003). Cynaropicrin (from the aerial parts of *Centaurothamnus maximus*, Compositae) showed *in vitro* cytotoxic activity against human cancer cell lines of malignant melanoma (SK-MEL), as well as against epidermoid (KB), ductal (BT-549) and SK-OV-3 carcinomas with IC_50_ values between 2.0 and 6.3 μg/ml (Muhammad et al., 2003). The cytotoxicity of cynaropicrin (from *Saussurea calcicola*, Compositae) was examined using a Sulforhodamin B Bioassay (SRB) against the five cultured human tumor cells: A549, SK-OV-3, SK-MEL-2, XF498 (CNS), and HCT-15. Cynaropicrin showed non-specific significant cytotoxicity against those human tumor cell lines with ED_50_ values ranging from 0.29 to 1.37 μg/ml (Choi et al., 2005). In another study, cynaropicrin (from the aerial parts of *Saussurea pulchella*, Asteraceae) exhibited a promising cytotoxicity against SK-MEL-2 and SK-OV-3 human tumor cell lines with ED_50_ values of 4.07 μM, and 7.42 μM, respectively. It showed also weak cytotoxicity against A549 and HCT (colon adenocarcinoma) with ED_50_ values of 24.51 and 12.13 μM, respectively. Doxorubicin was used as a positive control and its cytotoxicity against A549, SK-OV-3, SK-MEL-2, and HCT cell lines were ED_50_ 0.007, 0.056, 0.117, and 0.164 μM, respectively (Yang et al., 2008). Furthermore, cynaropicrin (from the aerial parts of *Centaurea omphalotricha*, Asteraceae) and cynaropicrin derivatives (3-acetyl cynaropicrin and 4′-acetyl cynaropicrin) were found to be cytotoxic compounds against human leukemia cell lines HL-60 and U937 with IC_50_ values of 2.0 ± 0.9 and 5.1 ± 0.4 μM, respectively (Kolli et al., 2012).

**Figure 3:**
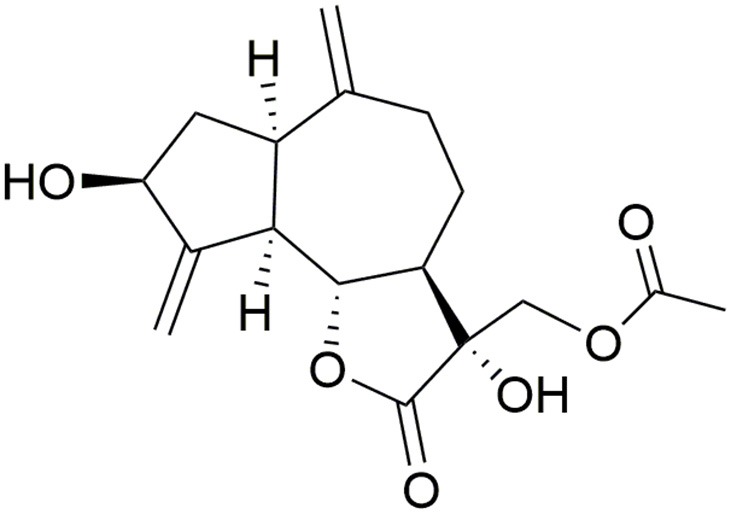
**Structure of 13-***O***-acetylsolstitialin A**.

Although cynaropicrin, being the major constituent of the edible plant artichoke, has a history of safe dietary use in humans, toxic effects were studied in some animal species. Ingestion of yellow star thistle (*Centaurea solstitialis*) by horses, but not cattle or sheep, produces Parkinsonism due to nigro-pallidal degeneration. The “chewing disease” or “yellow star thistle poisoning,” occurred in 1954 in central and northern California, was experimentally linked to the ingestion of large amounts of *C*. *solstitialis*. Thistle poisoning develops in horses within 1–3 months of feeding on the mentioned plant. It is characterized by facial muscle immobility, chewing problems and flicking of the tongue. This leads to inability to eat or drink normally, followed by hypokinesia and a lack of reactivity and eventually death. The dichloromethane extract of *C. solstitialis* was neurotoxic to neuronal cultures of fetal rat brain. Upon bioguided fractionation of the extract, 13-*O*-acetylsolstitialin A (Figure 3) and cynaropicrin exhibited neurotoxic activity in the test system using rat mesencephalic full culture where they caused a concentration-dependent reduction in the percentage of living cells with IC_50_ values of 3.6 and 3.0 μM, respectively (Wang et al., 1991; Cheng et al., 1992).

Applied on rabbit isolated aortic ring preparations, cynaropicrin was shown to induce toxic inhibition of smooth muscle contractility. The chemically-reactive α-methylenebutyrolactone function group is the pharmacophore moiety of the compound and is an essential functionality for smooth muscle inhibitory activity (Hay et al., 1994).

## Anti-inflammatory activity

TNF-α is a cytokine produced by many cells, especially lymphocytes and macrophages, which has an essential role in host chronic and acute inflammatory reactions. During inflammation, these cells are proliferated and activated by inflammatory signals (e.g., bacterial products such as lipopolysaccharide, concanavalin A, or phytohemagglutinin). Accordingly, different pro-inflammatory mediators are produced, such as cytokines (TNF-α, IL-1 and -6), eicosanoids (leukotriene B_4_ and prostaglandin E_2_), as well as reactive oxygen and nitrogen intermediates including nitric oxide (Cho et al., 2000). TNF-α is a monocyte-derived cytotoxin and cytokine that triggers the inflammatory reaction and hence its production deregulation is associated with autoimmune complications of human diseases such as rheumatoid arthritis, psoriasis, Alzheimer's disease, refractory asthma, cancer, and inflammatory bowel disease (IBD). It is implicated in tumorigenesis inhibition (TNF-α causes cytolysis of certain tumor cell lines) and viral replication and response to sepsis via IL1 & IL6 producing cells (Kriegler et al., 1988).

Cynaropicrin has potent suppressive effects on TNF-α and cytokine-induced neutrophil chemoattractant-1 and nitric oxide release, suggesting that cynaropicrin may be a useful agent toward acute and chronic inflammatory diseases. Cynaropicrin (from *Saussurea lappa*) strongly inhibited, at non-cytotoxic concentrations, the production of TNF-α from lipopolysaccharide-stimulated murine macrophage RAW264.7 cells, and U937 cells which are known producers of TNF-α (Cho et al., 1998). In a dose-dependent manner, cynaropicrin also potently inhibited the release of nitric oxide from lipopolysaccharide- and interferon-γ-stimulated RAW264.7 cells. Also in a dose-dependent manner, cynaropicrin suppressed lymphocytes proliferation from splenocytes and IL-2-sensitive cytotoxic T lymphocytes, CTLL-2 cells, stimulated by lipopolysaccharide, phytohemagglutinin, concanavalin A, and IL-2. Treatment with sulfhydryl (SH) compounds such as L-cysteine, 2-mercaptoethanol, and dithiothreitol abrogated all these inhibitory effects of cynaropicrin on TNF-α production and hence it was concluded that cynaropicrin may participate in the inflammatory response by inhibiting the production of inflammatory mediators and the proliferation of lymphocytes (Cho et al., 2000).

## Antiphotoaging and antioxidant activities

Photoaging is the main causative agent for the increased risk for skin cancer and the appearance of damaged skin. Aging of the skin is a complicated biological process, which is characterized by skin wrinkling, laxity, and pigmentation, and induced by several environmental and biological factors. The most common factor is the ultraviolet irradiation; the burning rays UVB (280–320 nm) makes up ~5% of UV light and UVB causes sunburn 1000 times more than UVA and is responsible for the photo-induced skin damage. UVB irradiation induces several intracellular signaling pathways leading eventually to the formation of ROS. The later, in turn, leads to the damage of vital macromolecules such as lipids, proteins, and nucleic acids in keratinocytes. Eventually, this will contribute to the process of photocarcinogenesis and photoaging. In addition, UVB irradiation induces the release of various pro-inflammatory cytokines in keratinocytes such as IL-6 and TNF-α which finally activate NF-κB (Fisher et al., 2002). Many researches showed that excessive activation of NF-κB has an essential role for skin photoaging and other disorders such as cancer, psoriasis, and rheumatism. NF-κB stimulation will increase the production of matrix metalloprotease-1 (MMP-1) and basic fibroblast growth factor (bFGF) which causes epidermal thickening, due to hyperproliferation, and pigment deposition, due to melanocyte proliferation, leading finally to photoaging. The UVB-induced skin photoaging processes are ascribable to the activation of NF-κB, which exist in epidermal keratinocyte and dermal fibroblasts (Balistreri et al., 2013). Therefore, NF-κB inhibitors will be novel ingredients in the anti-aging skin care treatments. Cynaropicrin is a very effective antiphotoaging agent acting by suppression of the NF-κB-mediated transactivation of bFGF and MMP-1 without significant cytotoxicity. Cynaropicrin prevented skin photoaging processes in an *in vivo* mouse model experiments (Tanaka et al., 2013).

Cynaropicrin is a very potent activator for AhR (aryl hydrocarbon receptor)–Nrf2 (nuclear factor E2–related factor 2)–Nqo1 (NAD(P)H:quinone oxidoreductase 1) pathways in normal human keratinocytes. In addition, it decreases the generation of ROS and the production of inflammatory cytokines in UVB-irradiated keratinocytes. Therefore, cynaropicrin could be applied to prevent UVB-induced photoaging. The cynaropicrin-induced AhR–Nrf2–Nqo1 activation was AhR- and Nrf2-dependent [Nrf2 is a key transcription factor that upregulates a series of antioxidative enzymes such as NAD(P)H:quinone oxidoreductase 1 (Nqo1)], as observed from that it was absent in keratinocytes transfected by siRNA (small interfering RNAs) against either AhR or Nrf2. In parallel with the activation of the AhR–Nrf2–Nqo1 system, cynaropicrin actively inhibited generation of ROS from keratinocytes irradiated with UVB in a Nrf2-dependent manner. Nqo1 is one of the key antioxidant enzymes that efficiently inhibit ROS production in keratinocytes. Cynaropicrin is a potent antioxidant since its EC_50_ and CC_50_ on Nqo1 induction was 0.89 ± 0.14 and 47.6 ± 2.8 μM, respectively, in keratinocytes. Cynaropicrin also inhibited the production of pro-inflammatory cytokines such as IL-6 and TNF-α from UVB-treated keratinocytes (Takei et al., 2015). In a placebo-controlled study with 8 volunteers, the artichoke leaf extract (contains 0.7% cynaropicrin) improved the facial pigmentation, conspicuous pores, and wrinkles after 8 weeks of intake through preventing the decline of dermal proteoglycan, which is important for water holding function (Takahashi, 2014).

The effect of cynaropicrin on the wasting syndrome (cachexia) and oxidative stress elicited by 2,3,4,7,8-pentachlorodibenzofuran (PenCDF) was evaluated in mice. Cynaropicrin has an ability to reduce oxidative stress caused by PenCDF by studying its effect on PenCDF-induced toxicity in C57BL/6J mice, a responsive strain to dioxins. Since 2,3,7,8-tetrachlorodibenzo-*p*-dioxin (0.1 mg/kg) induces hepatic ethoxyresorfin O-deethylase (EROD) activity in mice, however, this compound up to 20 mg/kg (p.o.) did not attenuate PenCDF-induced cachexia. On the contrary, PenCDF-induced oxidative stress was suppressed by cynaropicrin at the highest dose (20 mg/kg), although EROD activity was increased rather than reduced by cynaropicrin at lower doses (Yamada et al., 2015).

## Antibacterial activity

Cynaropicrin is a potent, irreversible inhibitor of the bacterial enzyme MurA which is of vital importance for bacterial cells since this enzyme is responsible for the first step in the cytoplasmic biosynthesis of peptidoglycan precursor molecules. Cynaropicrin covalently binds to the thiol group of Cys115 through Michael addition reaction. Bachelier et al. presented the first explanation of the antibacterial mode of action of sesquiterpene lactones on a molecular basis using *Escherichia coli*. Judging from the structure-activity relationships with other studied sesquiterpene lactones, the unsaturated ester side chain of cynaropicrin is of particular importance for the inhibition of MurA. In contrast, the α-methylene-γ-butyrolactone group and the exocyclic methylene moiety in the macrocyclic part of the cynaropicrin are less relevant. Concerning the binding mode, the ester side chain of cynaropicrin mimic the substrate phosphoenolpyruvate (PEP), whereas the macrocyclic part of the molecule is of minor importance (Bachelier et al., 2006).

## Anti-parasitic activity

The human parasite infections include deadly protozoal diseases, especially in tropical and subtropical developing countries. Human African trypanosomiasis (HAT) or sleeping sickness is caused by the protozoan parasite *Trypanosoma brucei* and is transmitted by blood-feeding tsetse flies (*Glossina* spp. Wiedemann) occurring in sub-Saharan Africa. The disease is fatal when left untreated. Currently, there are about 30,000 new HAT cases annually, and as many as 30 million people live in HAT endemic areas. In Central and Western Africa, about 95% of HAT cases are caused by *T. b. gambiense*, which causes a chronic form of sleeping sickness that can extend for months or years before the appearance of clinical symptoms. *T. b. rhodesiense* is endemic to Eastern Africa and causes the other 5% of cases and its infection causes an acute, more virulent form of HAT. A nonspecific malaise syndrome (the first stage) manifests both HAT forms, followed by the invasion of the parasites into the central nervous system (CNS) (the encephalitic stage) which triggers the progressive breakdown of neurological functions, including the disruption of the sleep cycle. Both HAT forms are life-threatening if not treated adequately (Zimmermann et al., 2012, 2014).

Cynaropicrin is the first plant-derived natural product with *in vivo* activity against *T. brucei*. It has reduced parasitemia in the murine model of trypanosomiasis and also it has potent antitrypanosomal activity *in vitro* (Zimmermann et al., 2012). Cynaropicrin (from the herb *Centaurea salmantica* L., Asteraceae) showed an *in vitro* inhibition of *T. brucei rhodesiense*. It causes a significant inhibition against *T. b. rhodesiense* and *T. b. gambiense* with IC_50_ values of 0.3 and 0.2 μM, respectively. However, it has a relatively moderate activity against *Trypanosoma cruzi* and *Plasmodium falciparum* with IC_50_ values of 4.4 and 3.0 μM, respectively. On day seven post-infection, I.p. administration of 2 × 10 mg/kg body wt/day in the *T. b. rhodesiense* STIB 900 acute mouse model (this model mimics the first stage of the HAT) resulted in a 92% reduction of parasitemia compared to untreated controls (Zimmermann et al., 2012; da Silva et al., 2013).

The antitrypanosomal activity of cynaropicrin is mediated by the depletion of intracellular glutathione (GSH) and trypanothione (T(SH)2) (which the trypanosomes depend on for redox regulation) as well as the inhibition of trypanosomal ornithine decarboxylase. This action was mediated by the cynaropicrin α,β-unsaturated methylene moiety which acts as Michael acceptor for (GSH) and trypanothione (T(SH)2). The analysis of this mechanism and the effects of cynaropicrin on enzymes of the T(SH)2 redox metabolism including glutathione-S-transferase, trypanothione synthetase, trypanothione reductase, and ornithine decarboxylase were established with UPLC–MS/MS analysis and the intra-cellular cynaropicrin, T(SH)2, GSH, as well as GS-cynaropicrin and T(S-cynaropicrin)2 adducts in intact *T. b. rhodesiense* cells were quantified. The cellular GSH and T(SH)2 pools were entirely depleted, within minutes of exposure to cynaropicrin, and the parasites entered an apoptotic stage and died. Cynaropicrin also inhibited the ornithine decarboxylase likewise the positive control eflornithine (Zimmermann et al., 2013).

**Figure 4:**
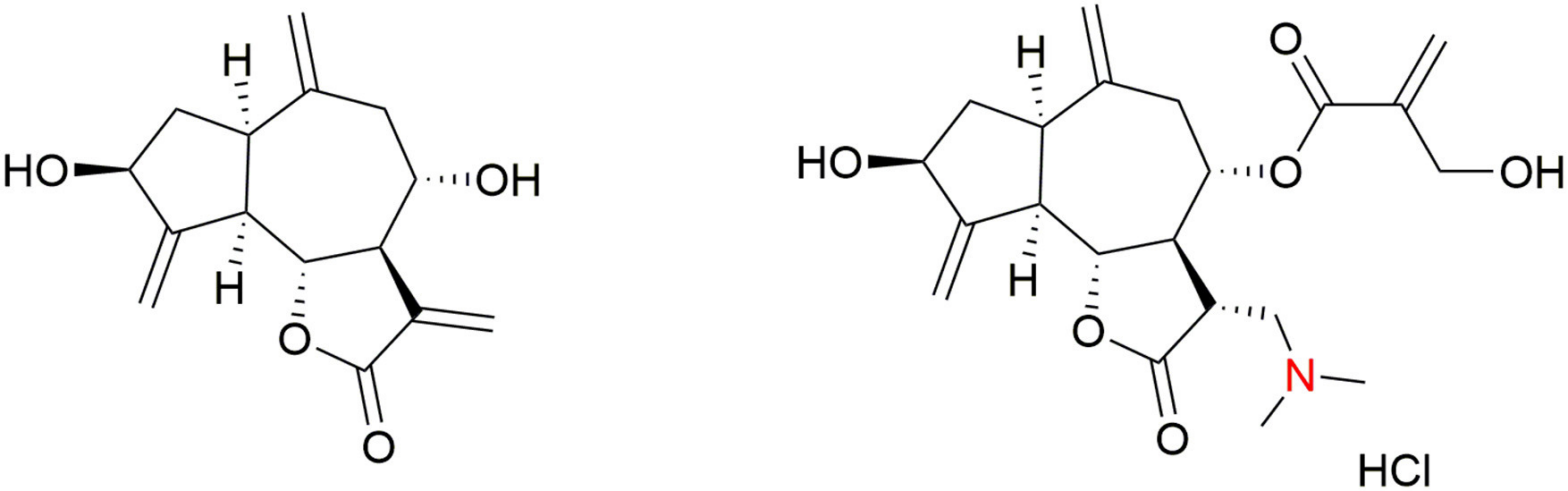
**Structures of deacylcynaropicrin and dimethylamino cynaropicrin derivative**.

The *in vitro* structure–activity-relationship (SAR) study of synthetic cynaropicrin derivatives against *T. brucei* was described. The side chain moiety of cynaropicrin plays a crucial role in its inhibitory activity. Removal of the 2-hydroxymethyl-2-propenoyl moiety of cynaropicrin (producing deacylcynaropicrin, Figure 4) resulted in a loss of toxicity toward *T. b. rhodesiense* and exhibited activity against *T. brucei* ten times less than that of cynaropicrin (Zimmermann et al., 2012).

Further derivatives were synthesized exploiting the hydroxyl groups in cynaropicrin and all of these compounds were tested for *in vitro* activity against *T. b. rhodesiense*. Based on the results of the SAR study, Usuki et al. concluded the following: “*(1) an acyl side chain at OH-8 is essential for antitrypanosomal activity and the 2-hydroxylmethyl-2-propenoic acid side chain affects SI value; (2) changing the hydrophilicity of the OH-3 substituent affects neither antitrypanosomal activity nor cytotoxicity; (3) changing the hydrophilicity of OH-19 group does not affect the antitrypanosomal activity, but does influence the cytotoxicity; (4) formation of an ester at the OH-19 position decreases both the antitrypanosomal activity and cytotoxicity; and (5) introduction of Ac groups at OH-19 and OH-3 positions leads to a decrease in both the antitrypanosomal activity and cytotoxicity”* (Usuki et al., 2014). These results suggest that derivatization of the two hydroxyl groups in cynaropicrin does not significantly affect the antitrypanosomal activity (Usuki et al., 2014). Additionally, many semi-synthetic sesquiterpene lactones and amino-sesquiterpene lactones were *in vitro* tested against *T. b. rhodesiense* and mammalian cancer cells (rat bone myoblast L6 cells). It was found that the α-methylene-γ-lactone moiety is necessary for both antitrypanosomal effects and cytotoxicity. Antitrypanosomal selectivity is facilitated by 2-(hydroxymethyl)acrylate (as in cynaropicrin) or 3,4-dihydroxy-2-methylenebutylate side chains, and by the presence of cyclopentenone rings. Semi-synthetic amino-cynaropicrin with dimethylamino group (Figure 4) was tested in the *T. b. rhodesiense* acute mouse model, where it showed reduced toxicity over cynaropicrin, but also lost antitrypanosomal activity (Zimmermann et al., 2014).

The trypanocidal activity of cynaropicrin was also evaluated against *Trypanosoma cruzi* (the causative agent of Chagas disease = American trypanosomiasis). The *in vitro* studies done by Schinor et al. showed that cynaropicrin (isolated from the Brazilian plant *Moquinia kingii*, Asteraceae) has an IC_50_ value of 93.5 μg/ml (Schinor et al., 2004). The *in vitro* studies done by Da Silva et al. showed that although cynaropicrin presented quite considerable trypanocidal effects *in vitro* (as effective as the control drug benznidazole), the treatment (once or twice a day) of *T. cruzi*-infected mice (up to 50 mg/kg/day) did not suppress parasitemia or protect against mortality induced by the Y and Colombiana strains compared to benznidazole (da Silva et al., 2013). In another study, the *in vitro* activity of cynaropicrin (from *Vernonia mespilifolia*, Asteraceae) against *Trypanosoma brucei rhodesiense, T. cruzi, Leishmania donovani*, and *P. falciparum* showed promising findings with IC_50_ values of 0.23, 5.14, 1.56, and 1.56 μM, respectively (Mokoka et al., 2011, 2013).

Cynaropicrin [from *Saussurea salsa (Pall.)* Spreng.] showed promising antiopisthorchiatic activity which has been studied *in vivo* on a model of opisthorchiasis in golden hamsters. Cynaropicrin produced a dose-dependent therapeutic effect comparable with that of the reference antischistosomal drug praziquantel (Drab et al., 2005).

## Antifeedant activity

Cynaropicrin is a promising antifeedant compound, which deserves further attention for development as an ecologically safe plant product for insect control and deterrent against herbivory. Cynaropicrin (from *Centaurea ptosimopappa* Hayek and *Trichlorepis glaberrima* DC, Compositae) showed a potent feeding deterrent activity against several species of Lepidoptera. Cynaropicrin proved to be a potent antifeedant on testing against 4th instar larvae of Bihar hairy caterpillar, *Diacrisia obliqua* Walker (*Lepidoptera: Arctiidae*) and the Erisilk worm *Philosamia ricini* Hutton (*Lepidoptera: Saturniidae*). The compound retained appreciable feeding deterrence up to two days from treatment (Bhattacharyya et al., 1995). Cynaropicrin (from aerial parts of *Rhaponticum pulchrum*) showed medium antifeedant activities (with an average index value activity of 66.6) against three species of stored product insect pests of *Sitophilus granarius* beetles, *Tribolium confusum* larvae and *Trogoderma granarium* larvae in no-choice and 2-choice feeding experiments (Cis et al., 2006).

## Gastric actions

Cynaropicrin possesses a marked effect on mucosal injuries, preventing acute gastritis. Using acute gastric mucosal injury in rats, the artichoke leaf extract exhibited an anti-gastritis action (gastric cytoprotective activity) which was attributed to its content of cynaropicrin, at least in part, by its gastric mucus glycoprotein-increasing action. Additionally, cynaropicrin increased the volume of gastric juice by stimulating the secretion of saliva and gastric juice secretion due to its bitter stomachic character (Ishida et al., 2010). Cynaropicrin is one of the main active choleretic ingredients in *Saussurea amara* where cynaropicrin at low and intermediate concentrations provoked a dose-dependent increase in bile flow in the perforated rat liver perfusion system which was even more than cynarin and apigenin-7-*O*-glucoside. Higher doses of pure cynaropicrin (20 mg/L) caused liver damage (Glasl et al., 2007). Overall, the beneficial gastric actions of moderate amounts of cynaropicrin that are present in artichoke leaf extracts may be the reason behind their massive and global use to ameliorate dyspeptic symptoms (Marakis et al., 2002).

Cynaropicrin (from the Brazilian *Cynara scolymus*) is a promising antispasmodic agent since it showed potent inhibitory effect on the contractile response elicited by acetylcholine on guinea-pig ileum in a noncompetitive and concentration-dependent manner, with an IC_50_ value of 0.065 mg/ml and having similar potency to that of papaverine. Cynaropicrin did not show any agonist effect, as it did not interfere with the basal tension of the preparations. In addition, after successive washings, the contractile response to agonists was restored. These findings are consistent with the popular use of artichoke as a home remedy for the treatment of gastrointestinal disturbances (Emendörfer et al., 2005). In the same context, another recent study showed that cynaropicrin has a weak *in vitro* acetyl cholinesterase inhibition with an IC_50_ value of 10 μg/ml and radical scavenging activity against 1,1-diphenyl-2-picryl-hydrazil (DPPH) radicals (Hegazy et al., 2016).

The different discussed activities of cynaropicrin including the model, dose and its source are summarized in Table 1.

**Table 1:** **Overview of reported activities of cynaropicrin, its sources, models used to detect the respective effects, and applied doses**.

**Activity**	**Model**	**Dose**	**Source of cynaropicrin**	**References**
Anti-HCV activity	*In vitro*, using hepatic tissues infected with different genotypes of HCV	EC_50_ from 0.4 to 1.4 μM for different genotypes of HCV	Wild Egyptian artichoke leaves, *Cynara cardunculus* L. var. *sylvestris* (Lam.) Fiori, Compositae (Asteraceae)	Elsebai et al., 2016b
Anti-hyperlipidemic activity	Olive oil-loaded mice	50 and 100 mg/kg, (2 h after olive oil administration)	Artichoke leaves, *Cynara scolymus* L.	Shimoda et al., 2003
Anti-tumor and cytotoxic activity	Quantification of major adhesion molecules (CD98 and CD29) in macrophages, using a quantitative aggregation assay established with U937 cells	IC_50_ 2.98 and 3.46 μM, respectively	Cynaropicrin (purity: 97%) was purified from roots of *Saussurea lappa*	Cho et al., 2004a
	Leukocyte cancer cells (e.g., lymphoma or leukemia)	Cynaropicrin dose-dependently decreased the viability of U937, Eol-1 and Jurkat T cells with IC_50_ values of 3.11, 10.9 and 2.36 μM, respectively	Roots of *Saussurea lappa*	Cho et al., 2004b
	Human gastric adenocarcinoma (AGS) cells	IC_50_ 0.68 μg/ml	The aerial part of the Mongolian medicinal plant *Saussurea salicifolia*	Kang et al., 2007
	IL-6-inducible and constitutive STAT3 activation in THP- 1 cells and the cell line DU145	IC_50_ of 12 μM	Cynaropicrin was purchased from PhytoLab (Vestenbergsgreuth, Germany)	Butturini et al., 2013.
	Homozygotization Index (HI) test on two diploid strains of *Aspergillus nidulans*: UT184//UT448	Cells exposed to the drug at a concentration of 25 μg/ml	From *Moquinia kingii* (H. Robinson) Gamerro (Vernonieae, Compositae)	Salvador et al., 2008
	Solid and ascites tumors (S-180 sarcoma and Ehrlich carcinoma)	IC_50_ values of 2.5 and 2.4 μg/ml for cynaropicrin	Isolated from the Tibetan plant *Saussurea eopygmaea*, Compositae	Zong et al., 1994.
	SK-OV-3, LOX-IMVI, A549, MCF-7, PC-3, and HCT-15 cell lines	IC_50_ values between 1.1 and 8.7 μg/ml	From the flowers of *Hemisteptia lyrata* Bunge	Ha et al., 2003.
	Human cancer cell lines of malignant melanoma (SK-MEL), epidermoid (KB), ductal (BT-549) and SK-OV-3	IC_50_ values between 2.0 and 6.3 μg/ml	From the aerial parts of *Centaurothamnus* maximus, Compositae	Muhammad et al., 2003.
	Sulforhodamin B Bioassay (SRB) against five cultured human tumor cells: A549, SK-OV-3, SK-MEL-2, XF498 (CNS), and HCT-15	ED_50_ values ranging from 0.29 to 1.37 μg/ml	From *Saussurea calcicola*, Compositae	Choi et al., 2005.
	SK-MEL-2 and SK-OV-3 human tumor cell lines	ED_50_ values of 4.07 μM, and 7.42 μM, respectively	From the aerial parts of *Saussurea pulchella*, Compositae	Yang et al., 2008
	Human leukemia cell lines HL-60 and U937	IC_50_ values of 2.0 ± 0.9 and 5.1 ± 0.4 μM/L	From the aerial parts of *Centaurea omphalotricha*, Compositae	Kolli et al., 2012
Anti-inflammatory activity	Murine macrophage RAW264.7 cells, and U937 cells	IC_50_ on TNF-α production was 2.86 μg/ml	Roots of *Saussurea lappa*	Cho et al., 1998
	The release of TNF-α and NO from either differentiated U937 cells or RAW264.7 cells activated by lipopolysaccharide and interferon-γ	IC_50_s between 1.1 and 8.24 μM	Roots of *Saussurea lappa*	Cho et al., 2000
Antiphotoaging and antioxidant activities	Melanocytes	The cells were pretreated with cynaropicrin 2, 4 or 8 μM	Artichoke *(Cynara scolymus)*	Tanaka et al., 2013
	Keratinocytes	EC_50_ and CC_50_ on Nqo1 induction was 0.89 and 47.6 μM, respectively	Artichoke *(Cynara scolymus)*	
	PenCDF-induced toxicity in mice	20 mg/kg	Artichoke *(Cynara scolymus)*	Yamada et al., 2015
Antibacterial activity	The MurA enzymes of *E. coli* K12 and *P. aeruginosa* PAO1293	IC_50_ = 12.5 μM for E. coli MurA, at 12 nM. IC_50_ = 12.1 μM for *P. aeruginosa* MurA, at 150 nM	Leaves of *Cynara scolymus* L. (Caelo, Hilden, Germany)	Bachelier et al., 2006
Anti-parasitic activity	Murine model of trypanosomiasis	Inhibition against *T. b. rhodesiense* and *T. b. gambiense* with IC_50_ values of 0.3 and 0.2 μM, respectively	From the herb *Centaurea salmantica* L., Compositae	Zimmermann et al., 2012; da Silva et al., 2013
	Blood collected from *Trypanosoma cruzi*-infected mice	IC_50_ value of 93.5 μg/ml	From the Brazilian plant *Moquinia kingii*, Compositae	Schinor et al., 2004
	*Trypanosoma brucei rhodesiense, T. cruzi, Leishmania donovani*, and *Plasmodium falciparum* in rat myoblast cells (L6-cells) as in Mokoka et al., 2011	IC_50_ values of 0.23, 5.14, 1.56, and 1.56 μM, respectively	From *Vernonia mespilifolia*, Compositae	Mokoka et al., 2013
	Opisthorchosis model in golden hamsters	At a dose of 0.8 mg/kg, the samples taken from hamsters treated with cynaropicrin showed no fluke eggs under the microscope on the 7th day	The aerial parts from *Saussurea salsa* (Pall.) Spreng.	Drab et al., 2005
Antifeedant activity	4th instar larvae of Bihar hairy caterpillar, *Diacrisia obliqua* Walker and the Erisilk worm *Philosamia ricini* Hutton	The compound was tested at 0.02, 0.05, 0.25, 0.5 and 1% cynaropicrin in acetone. EC50 0.01 and 0.41, respectively	From *Centaurea ptosimopappa* Hayek and *Trichlorepis glaberrima* DC, Compositae	Bhattacharyya et al., 1995
	Three species of stored product insect pests; *Sitophilus granarius* beetles, *Tribolium confusum* larvae and *Trogoderma granarium* larvae	Average index value activity of 66.6	From aerial parts of *Rhaponticum pulchrum*	Cis et al., 2006
Gastric actions	Male Sprague-Dawley strain SPF rats (Nippon SLC, Shizuoka, Japan)	Cynaropicrin at an oral dose of 5 mg/kg markedly prevented the mucosal injury by 98%	Artichoke (*Cynara Scolymus* L.) leaf extract	Ishida et al., 2010)
	Male Sprague-Dawley rats	At low doses, cynaropicrin is one of the main active choleretic principles in *S. amara*	Aerial parts of *Saussurea amara*, Compositae	Glasl et al., 2007
	Guinea-Pig ileum	IC_50_ value of 0.065 mg/ml	From Brazilian *Cynara scolymus*	Emendörfer et al., 2005

## Biosynthesis

The highest concentration of cynaropicrin is found in leaves (9.6 ± 0.4 mg.g^−1^ DW), regardless of their stage of development, suggesting they are likely the place where cynaropicrin is synthetized and stored; cynaropicrin predominantly accumulates in the trichomes. Other plant organs such as the receptacle show a progressively decreasing content of cynaropicrin during the inflorescence development. At the same time, for stems, roots and bracts, cynaropicrin was undetectable (Eljounaidi et al., 2015).

**Figure 5:**
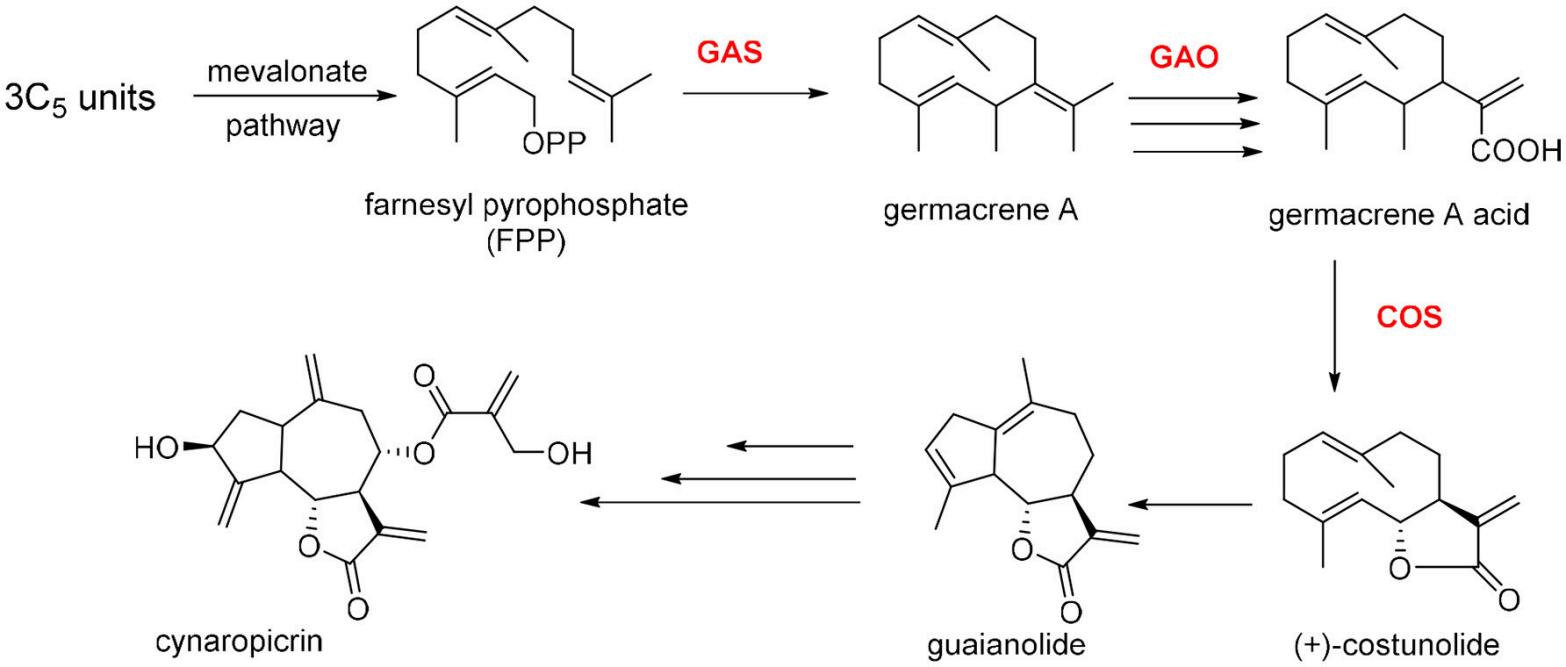
**Proposed biosynthetic pathway for cynaropicrin**.

Cynaropicrin is formed from three isoprene C_5_ units and synthesized via mevalonate pathway. Cynaropicrin is likely derived from the common precursor costunolide, the product of germacrene A, by the action of two cytochrome P450 enzymes (Cytochrome P450s from *C. cardunculus* CYP71BL5 and CYP71AV9, catalyze specific hydroxylations in the sesquiterpene lactone biosynthetic pathway). Therefore, germacrene A biosynthesis is assumed to be the first committed step in the biosynthesis of cynaropicrin and generally for guaianolide sesquiterpene lactones (Figure 5). Cyclization of farnesyl pyrophosphate (FPP) is catalyzed by (+)-germacrene A synthase (GAS) to afford germacrene A. The latter is subjected to three oxidation steps catalyzed by cytochrome P450 germacrene A oxidase (GAO) to afford the corresponding acid. The acid is then hydroxylated by (+)-costunolide synthase (COS) into an unstable intermediate that undergoes a non-enzymatic lactonization yielding costunolide (“olide” refers to lactone group). The genetic mapping and characterization of the globe artichoke (+)-germacrene A synthase gene, encoding the first dedicated enzyme for biosynthesis of cynaropicrin was also reported in this study (Menin et al., 2012; Eljounaidi et al., 2014, 2015).

The synthesis of deuterated cynaropicrin at the side chain was achieved via esterification of hydrolyzed cynaropicrin with deuterated side chain (Sato et al., 2015).

## Sources of cynaropicrin

Sesquiterpene butyrolactones are natural products widely distributed in the Asteraceae (Compositae) family of flowering plants; cynaropicrin is a chemotaxonomic marker of artichoke plant species (Chaturvedi, 2011) where it was isolated, among others mentioned above, from different *Cynara* plants such as the Italian and Polish *C. scolymus* (Samek et al., 1971; Barbetti et al., 1993), the aerial (flowering) parts of the Japanese *C. cardunculus* (Shimizu et al., 1988), lipophilic extracts of *C. cardunculus* L. var. *altilis* (DC) (Ramos et al., 2013), and *C. humilis* (Reis et al., 1992).

Cynaropicrin has been also reported from the following botanical sources: The Chinese plant *Saussurea katochaete* (Asteraceae) (Saito et al., 2012), the aerial parts of *Saussurea lipshitzii* collected in South Gobi (Todorova et al., 1991), the aerial parts of *Saussurea amurensis* (Sham'yanov et al., 1988), *Saussurea affinis* (Das et al., 1983), *S. salicifolia* (Dudko and Rybalko, 1982), *Saussurea costus* (Pandey et al., 2007), and *Saussurea alata* (Ren et al., 2007). The aerial parts of *Centaurea scoparia* (Youssef and Frahm, 1994), *Centaurea ptosimopappoides* (Öksüz and Serin, 1997), *Centaurea bella* (Daniewski and Nowak, 1992), *C. solstitialis* (Merrill and Stevens, 1985), *Centaurea pannonica* (Heuff.) (Milošević Ifantis et al., 2013), the endemic Turkish plant *Centaurea hermannii* (Öksüz et al., 1994), the flowers of the endemic Turkish plant *Centaurea helenioides* Boiss. (Yayli et al., 2006), *Centaurea scabiosa* L. (Kaminskii et al., 2011), *Centaurea phaeopappoides* and *C. thracica* (Nowak et al., 1989), *Centaurea americana* (Ohno et al., 1973), *Centaurea ragusina* L. subspecies ragusina growing in Egypt (Mahmoud et al., 1986), *Centaurea canariensis* (Gonzalez et al., 1978), *Centaurea repens* (Stevens, 1982), *Centaurea kotschyi* (Oksuz and Putun, 1983), *Centaurea arguta* (Gadeschi et al., 1989); the roots of the Chinese plant *Vladimiria denticulate* (Jinyun et al., 2002), the aerial parts of *Leuzea rhapontica helenifolia* (Nowak et al., 1988), *Leuzea carthamoides* DC (Sovová et al., 2008), *Acroptilon repens* (Zhao et al., 2006), *Cheirolophus* species (Marco et al., 1994), from three species of *Cheirolophus* endemic to the Canary Islands (Gonzalez et al., 1993), some species of subtribe *Centaureinae Dumort* (Nowak et al., 1986), *Volutaria abyssinica* (Marzouk, 2015), the aerial parts of three species of the genus *Cousinia* (Marco et al., 1993), the aerial parts of *Cousinia adenostica* (Rustaiyan et al., 1987), *Aegopordon berarioides* (Izaddoost et al., 1985), *Grossheimia macrocephala* (Barbetti et al., 1985), *Amberboa divericata* (Rojatkar et al., 1997), *Amberboa ramose* (Harrison and Kulshreshtha, 1984), *Amberboa tubuliflora* (Ahmed et al., 1990), *Volutarella divaricata* (Forgacs et al., 1981), from *Chartolepis intermedia, C. glastifolia, C. biebersteinii*, and *C. pterocaula, Vernonia glutinosa* (Rakotoarimanga et al., 1992), *Brachylaena* species (Zdero et al., 1991), and *Artemisia xerophytica* (Tan et al., 1991).

## Discussion and conclusions

Cynaropicrin has multi-medicinal potential therapeutics evidenced not just by the ample scientific literature describing it, but also by the plenty number of patent applications (http://www.thegoodscentscompany.com/data/rw1701261.html, accessed 28-2-2016). The most outstanding activity recently discovered for cynaropicrin is the activity against HCV where it presents a very attractive pan-genotypic anti-HCV natural product. It showed potent and broad spectrum potential against the major genotypes of HCV by inhibiting viral cell entry.

The discovery of novel anti-HCV entry inhibitors such as cynaropicrin could help to develop preventive therapies/measures against hepatitis C where immunization against HCV is at present unavailable due to the highly mutable nature of the virus. Important applications of entry inhibitors include the prevention of recurrence in the new liver in HCV-patients after liver transplantation. In patients with HCV-related end-stage liver diseases undergoing liver transplantation, re-infection of the graft is universal and characterized by accelerated progression of liver diseases. Entry inhibitors may be effective especially in these patients undergoing violent re-infection of HCV into hepatocytes (Nakajima et al., 2013).

From the histo-pharmacology of cynaropicrin action, it seems that most of its pharmacological effects are convergent to support the anti-HCV activity, as follows:

(1) Cynaropicrin was found to suppress hyperlipidaemia as mentioned above (Shimoda et al., 2003). The lipids are important factors for the life-cycle of HCV where the virus replication and assembly is dependent on host cell lipid metabolism. HCV attaches itself to lipids and very-low-density lipoproteins after its release from the infected cells. It will then circulate in the blood in the form of triglyceride-rich particles (Burlone and Budkowska, 2009). It was also demonstrated that HCV-core protein (C), and the non-structural proteins NS2 and NS5A of HCV induce lipid accumulation in the liver cells resulting in steatosis, fatty liver and potential progression of liver disease and associated with hepatic inflammation and fibrosis. Therefore, it is important to lower increased cholesterol and triglycerides concentrations in HCV-infected patients (Kim et al., 2009).

(2) Cynaropicrin is a potent antioxidant (Takei et al., 2015; Yamada et al., 2015) and hence it can play a supportive role for liver in different hepatic diseases. Oxidative stress plays an important role in the pathogenesis of chronic hepatitis C. Oxidative stress occurs early during HCV infection and increases with disease progression and severity and hence the infected patient with HCV is usually being advised to take antioxidants such as vitamins C, D, and E, which are the most investigated as antioxidant therapy in chronic liver diseases (Milliman et al., 2000).

(3) Cynaropicrin is a potent inhibitor of TNF-α action which is a major mediator of the inflammatory process in HCV patients. Serum levels of TNF-α have been correlated with elevated alanine aminotransferase (ALT) and increased severity of fibrosis in HCV patients. Patients receiving interferon showed decreased TNF-α concentration (Milliman et al., 2000). It is also suggested that the activation of the NF-κB and AKT pathways are involved in increased HCV replication (Brenndörfer et al., 2009).

(4) Since cancer is the result of a deregulation of multiple signaling pathways and cynaropicrin elicit multi-targeted activities, this natural product could hold a great potential for treating human tumors. Cynaropicrin having significant antitumor activity and it may be served as potential cancer chemopreventive lead drug for prevention or treatment of human cancers (Kang et al., 2007). Human infection with HCV is currently recognized as the leading cause of the severe complications such as HCC which demands liver transplantation (Ishida et al., 2014) since HCC is the major histological subtype of primary liver cancer (Hu et al., 2013). A total of ~27% of individuals that develop liver cirrhosis and HCC worldwide arise in HCV-infected people (Ishida et al., 2014). It appears to be the major causative factor responsible for the recent doubling of HCC which was estimated to result in ~10,000 deaths in the United States only in the year 2011 (Gonzalez et al., 2009; Ibrahim et al., 2013). Cynaropicrin has anti-inflammatory, anti-tumor, TNF-α inhibitory, and antioxidant activities and hence it can also prevent the progression of HCC. Indeed, most natural agents do not induce a high level of toxicity and target multiple signaling pathways involved in cell growth, invasion, apoptosis, angiogenesis and metastasis (Pulito et al., 2015).

Cynaropicrin as a potential therapeutic compound is having many other advantages:

(1) The cynaropicrin is one of the main active constituents of artichoke; the therapeutic effects of the latter have been observed in several clinical trials (Giacosa et al., 2015) and it has recently attracted attention because of its well-proven safety at concentrations currently employed in alcoholic beverages (Cravotto et al., 2005). Another clinical study recommended artichoke extract for treating hyperlipoproteinemia and, thus, prevention of atherosclerosis and coronary heart disease and showed that there were no drug-related adverse events indicating an excellent tolerability of artichoke extract (Englisch et al., 2000). Artichoke is a fundamental component of Mediterranean diet and is available globally in all markets (Pulito et al., 2015). (2) The compound is water-soluble and hence therapeutic injections can be formulated which will decrease the amount of dosage form and shorten the duration of therapy and thus reducing putative side effects.

(3) The compound has a simple structure and hence it can be synthesized and marketed with affordable prices.

The final conclusion is that cynaropicrin is a very effective and promising natural product for hepatitis C virus infection and a prospective new drug lead, since it has a direct action on HCV by cell entry inhibition and cell-cell coinfection inhibition and also it may exert inhibitory effects on HCV replication via suppression of one or more signaling pathways related to ROS, Akt, NF-κB, and STAT3. Further in-depth studies including clinical trials are needed to fully evaluate its value for HCV treatment. Thus, cynaropicrin represents a natural product with an excellent therapeutic potential that can be considered as one illustrative example in line with the re-emerging notion that medicinal plants are expected to be a continues magnificent source of molecules with therapeutic potential (Atanasov et al., 2015).

## Author contributions

ME has written the first draft of the manuscript. AM and AA revised and improved the first draft. All authors have seen and agreed on the finally submitted version of the manuscript.

### Conflict of interest statement

The authors declare that the research was conducted in the absence of any commercial or financial relationships that could be construed as a potential conflict of interest.
